# Improving an endangered marine species distribution using reliable and localized environmental DNA detections combined with trawl captures

**DOI:** 10.1038/s41598-025-95358-3

**Published:** 2025-04-08

**Authors:** Marion Chevrinais, Audrey Bourret, Geneviève Côté, Geneviève Faille, Nellie Gagné, Geneviève J. Parent

**Affiliations:** 1 Fisheries and Oceans Canada, Maurice Lamontagne Institute, Mont-Joli, QC Canada; 2 Fisheries and Oceans Canada, Gulf Fisheries Centre, Moncton, NB Canada

**Keywords:** Groundfish, Traditional methods, Biomonitoring, Genomics, Molecular detections, Marine biology, Conservation biology

## Abstract

The description of marine fish distributions generally relies on trawl survey observations. For rare species, sporadic catches necessitate the combination of multiannual trawl surveys to accurately describe the distribution, limiting short term monitoring. Recent studies suggest that combining traditional capture methods and environmental DNA (eDNA) detections enhance rare species’ occurrence. In this study, the reliability and localization of eDNA detections (single- and multi-species) of an endangered marine species, the Atlantic wolffish *Anarhichas lupus*, was assessed during fine scale surveys. eDNA was detected at all six stations sampled with Niskin bottles over caves housing one or two *A. lupus*. Detections from samples collected with syringes by divers along a 15 m transect perpendicular to each cave were limited to the fish cave entrance. Trawl-captures and eDNA detections were then combined to test if the species distribution is improved for broad scale surveys. The station-based frequency of species occurrence was 13% with trawl captures and increased to 23% when combined with eDNA detections. Single-species detections were generally more sensitive than multi-species detections. Our results showed that a rare marine species distribution improves combining traditional methods and eDNA detections in oceanographic surveys. Strategies for integrating optimal eDNA detections in marine surveys are discussed.

**Supplementary Information:**

The online version contains supplementary material available at 10.1038/s41598-025-95358-3.

## Introduction

Most marine fish distributions are currently described and monitored using various direct observations including trawl captures, videos, and diving. Trawl survey is the main traditional method used to survey marine fish presence and abundance but has the disadvantage of being destructive and unsuitable for some habitats such as rocky bottoms^1^. Underwater videos and diving are non-destructive methods but are expensive on large scales, usually survey much smaller areas than trawl, and can be limited to shallow habitats^2^. All those direct observation methods show imperfect detectability, and a combination of methods can be more effective to estimate reliably marine species distributions^2^.

The detection of environmental DNA (eDNA) to infer species presence is a promising method to survey species distribution which has the advantage of being non-destructive^3^. The detection of species eDNA is based on the capture of DNA “from an environmental sample without any attempt to isolate the organism(s) from which the DNA derives”, and detection of the species to infer species presence^4^. Direct observations and eDNA detections methods are often compared to understand how concordant species occurrence are using either method with a wide extent of results^5–9^.

Some studies suggest similar results when characterizing the distribution and abundance of relatively abundant fish species with eDNA and acoustic-trawl estimates or eDNA and trawl captures estimates^10,11^. Those results are of primary interest for fisheries management science. However, the complementarity between eDNA and traditional methods is more commonly observed for low abundance or rare species^12^. Yet, skepticism towards the use of eDNA for conducting fish occurrence surveys is often expressed, primarily due to the potential for eDNA to disperse over extensive distances. Multiple recent studies have investigated this important question, and their results suggest that eDNA detections in different marine coastal environments may disperse from 100 m to 1.5 km^13–16^. In these studies, multiple specimens in cages were used to assess the dispersion of eDNA. For instance, DNA dispersion was studied using 49 striped jack (*Pseudocaranx dentex*)^15^ or 46 million juvenile Atlantic salmons (*Salmo salar*)^13^. However, these studies do not mimic the natural distribution and abundance of rare species.

The Atlantic wolffish *Anarhichas lupus* is distributed patchily in rocky coastal habitats in northern latitudes of the Atlantic Ocean. The species also inhabits caves with one or two individuals per cave^17^, limiting the production and dispersion of eDNA. This species suffered from a steep decline in density in some parts of its distribution range such as in the Gulf of St. Lawrence^18^. The species has been assessed as “special concern” by the Committee on the Status of Endangered Wildlife in Canada^18–20^ since 2001, “species of concern” by the National Marine Fisheries Service of United States of America since 2009^21^, and “endangered species” by the Baltic Marine Environment Protection Commission since 2007^22^. The rarity and the geographic isolation of animals in rocky habitats in the Gulf of St. Lawrence (Canada) provides additional challenges to study its distribution, and alternative methods could fill knowledge gaps.

Multi-species and single-species assays for eDNA detections are used to survey *A. lupus*. Multi-species assays amplify interspecific conserved regions (i.e., primers binding to sequences common to multiple taxa) and provide diversity detections for taxonomic groups. Multi-species assays amplifying the mitochondrial 12 S gene are often used for fish detections^23^. However, 12 S assays cannot discriminate species from the *Anarhichas* genus due to haplotype sharing^24^. The incapacity to discriminate *A. lupus* eDNA from that of *Anarhichas minor* and *Anarhichas denticulatus* in areas where the three species are potentially present could lead to an overestimation of *A. lupus* occurrences. In the Gulf of St. Lawrence, *A. minor* and *A. denticulatus* are classified as “threatened” species by COSEWIC^25^ and have rarer occurrences than *A. lupus*. Thus, it is highly probable that the majority of *Anarhichas* eDNA detections with 12 S assays would correspond to *A. lupus* DNA. Alternatively, a single-species assay was developed for *A. lupus*^26^ using probe-based quantitative PCR (qPCR) and allows for specific and highly sensitive results which is appropriate for the detection of low-abundance species^27^.

Our study was designed to characterize the usefulness of eDNA detection to improve a rare species distribution using single- and multi-species detection approaches. We first used three experimental designs on a fine scale natural environment to characterize the reliability of *A. lupus* eDNA detection by estimating the detection rate and the localization of eDNA detections. All these experimental designs were conducted in the Banc-des-Américains Marine Protected Area (BDA-MPA), within the Gulf of St. Lawrence. We then compared and combined eDNA detections and trawl-captures to assess possible improvements to the species distribution in a broad scale experimental design, the Estuary and the Gulf of St. Lawrence. The last objective of this study was to assess the sensitivity of single- and multi-species assays amplifying the DNA of *A. lupus* or *Anarhichas* spp. with experimental designs at the fine and broad scales.

## Materials and methods

### Sampling events

**Fig. 1:**
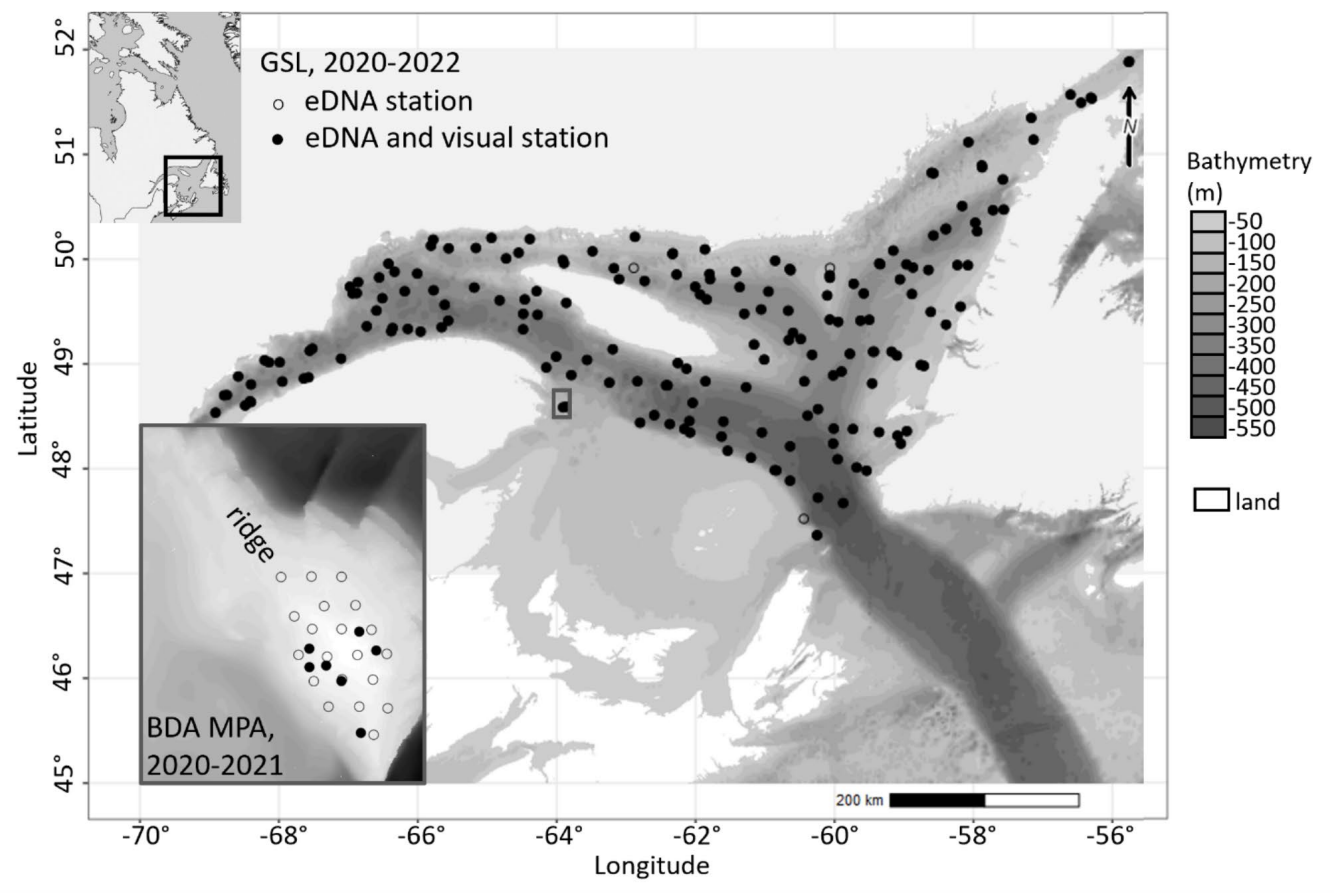
Study area at the broad scale with low density sampling in the Estuary and Gulf of St. Lawrence (GSL) and at the fine scale with high density sampling in the Banc-des-Américains Marine Protected Area (BDA MPA). The total number of eDNA samples collected at the broad and fine scales were of 188 and 78, respectively. Geographic reference system: NAD83 (CSRS) Quebec Lambert, EPSG 6622. Raster files were sourced from the National Oceanic and Atmospheric Administration (Bathymetric Data Viewer) for GSL and the Canadian Hydrographic Service (Canadian Hydrographic Service Non-Navigational (NONNA) Bathymetric Data - Open Government Portal) for BDA-MPA.

Surveys (sampling events) occurred in summers 2020, 2021, and 2022. Surveys were conducted at a fine scale on the ridge of the Banc-des-Américains Marine Protected Area (BDA MPA) which is ~ 3 km^2^ and about 15–60 m deep. In this area, the sporadic presence of *Anarhichas lupus* was confirmed during dives at 15–30 m depths in 2014^28^ (Fig. 1).

The ridge of the BDA-MPA is a highly dynamic system^28^. It is affected by the Gaspé Current, the most significant current in the Gulf of St. Lawrence, as well as by high-frequency internal wave trains with oscillations about 10 m in amplitude and reaching a depth of 60 m^28,29^. A first vessel-based survey with a matrix sampling design was conducted in July 2020 to assess for the frequency of occurrence of *Anarhichas lupus* in the area solely using environmental DNA (eDNA), herein designated BDA-matrix. A second vessel-based survey was conducted in June 2021 to assess *A. lupus* detection rate collecting eDNA over *A. lupus* caves, confirmed by SCUBA diving, and herein designated BDA-caves. The third survey was diving-based and occurred in June 2021 with perpendicular horizontal transects from *A. lupus* caves, to assess the dispersion of eDNA in a highly dynamic system, herein designated BDA-diving transect.

Sampling at broad scale took place during the Fisheries and Oceans Canada (DFO) annual ecosystemic surveys in 2020, 2021, and 2022, covering a large study area of more than 100,000 km^2^ in the Estuary and Gulf of St. Lawrence (GSL, Fig. 1) and sampling depths between 30 and 525 m. Water samples and bottom-trawl captures of *A. lupus* were obtained from these vessel-based surveys using a stratified random sampling design. Each bottom-trawl lasted 15 min (more details in^30–32^). Specimens of *A. lupus* trawl-captured were counted and the biomass was estimated^30–32^; (Supplementary Table S1).

### Water collection, filtration and preservation

Any equipment in contact with water before and after sampling was decontaminated using a 0.6% sodium hypochlorite solution, and gloves were changed between samples.

**Fig. 2:**
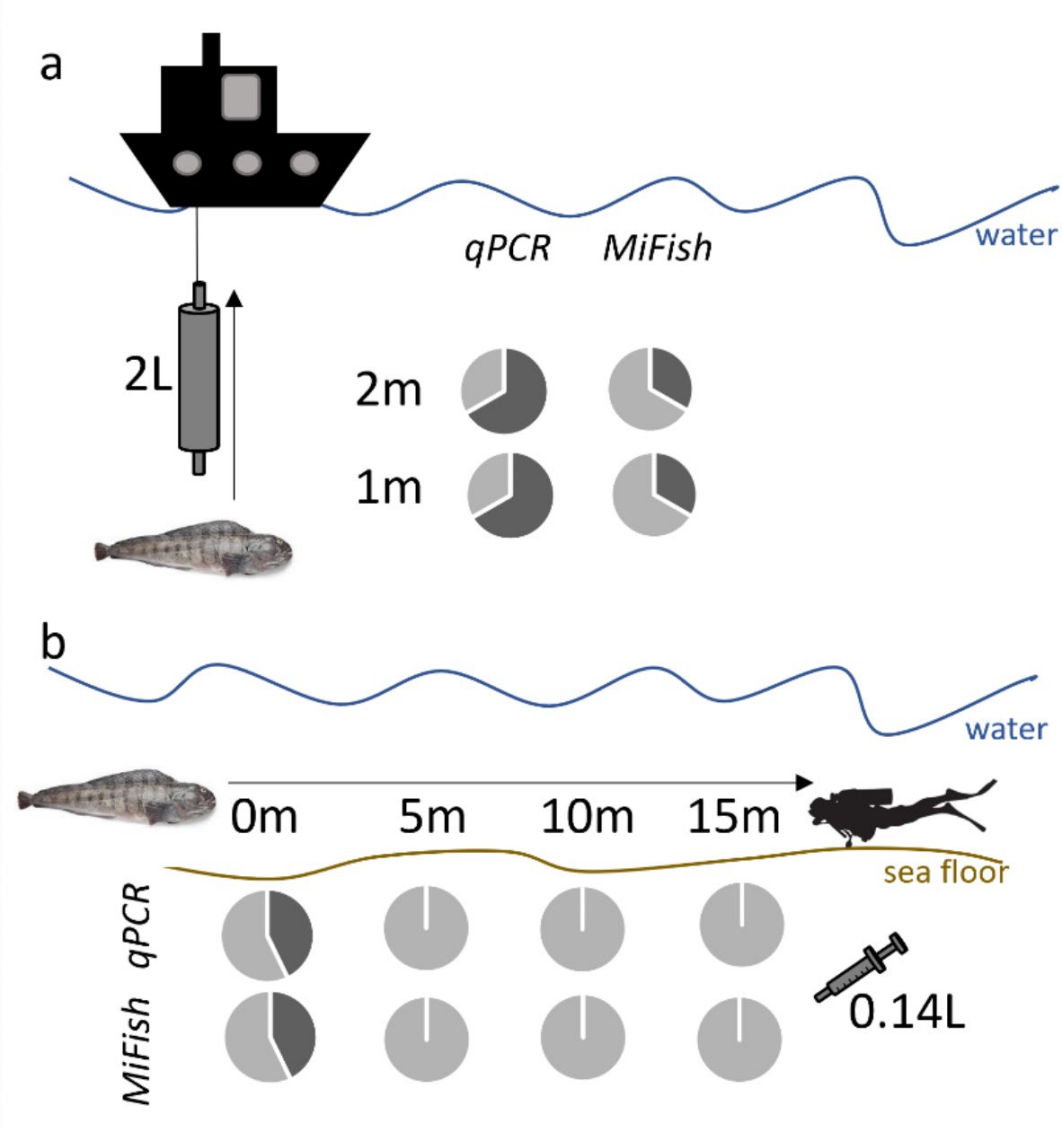
Fine scale experimental designs and eDNA detections of the 2021 survey in Banc-des-Américains Marine Protected Area (BDA-MPA). Water was collected either (**a**) with Niskin bottles from a vessel over Atlantic wolffish caves confirmed by a diver (BDA-caves), or (**b**) with syringes collected along a 15 m transect by divers (BDA-diving transect). Dark grey in pie charts represents the proportion of stations (N_total_ = 6 in a, 7 in b) with positive DNA detections of *Anarhichas lupus* (qPCR) or *Anarhichas* spp. (metabarcoding with MiFishU) at two depths (in a) or along a perpendicular transect (in meters, in b) from *A. lupus*. The combination of eDNA detection results at both depths or along a transect with either approach (qPCR or MiFishU metabarcoding) are presented in Fig. 3b.

For BDA-matrix and BDA-caves, two water samples of 2 L were collected per sampling station, i.e., at 1 and 2 m above the sea floor (Fig. 2a; Table 1). Water samples were either filtered on the vessel (BDA-matrix) or conserved on ice and filtered within 6 h post collection (BDA-caves) with a Smith-Root filter pack. At the broad scale, a single water sample of 2 L was collected per station at 1 m above the sea floor, and filtered on the vessel with a Smith-Root filter pack, except in 2020 where water was frozen at -40 °C until filtration at the Maurice Lamontagne Institute (MLI, Table 1). Filters from surveys were preserved either with the Smith-Root self-preserving filter pack, at -40 °C, or in silica beads until DNA extraction (Table 1).

For the BDA-diving transect survey, water samples were collected using a single 140 mL syringe, one at each of the 4 distances on a perpendicular horizontal transect from the entrance of the *A. lupus* cave, i.e., 0, 5, 10, 15 m (Table 1; Fig. 2b). Water samples from syringes were filtered with a Swinnex Filter Holder (MilliporeSigma, Darmstadt, Germany) containing a filter and preserved in silica beads until DNA extraction (see Table 1 for more details).

In all surveys, field negative controls were obtained by filtering (for the eDNA sampler system and syringes) or transferring between two bottles in the field (for samples preserved frozen) Milli-Q^®^ water (MilliporeSigma, Darmstadt, Germany). The volume of Milli-Q water used, and the preservation method of the filters followed the one used during the specific survey (Table 1).

**Table 1:** Sampling sites metadata and samples laboratory treatment for each survey (see Figs. 1, 3 and 5 for geographic locations of sites and supplementary material for details).

Surveys	Location	Year	Survey type	Number of stations	Samples per station	Volume (L)	Filtration equipment	Filter, pore size (µm)	Filter preservation	DNA extraction	Elution volume (µL)	qPCR per sample
BDA-matrix	Banc-des-Américains Marine protected Area	2020	vessel	20	2	2	E	PES, 1.2	Self- preserving filter pack	BTT	80	3
BDA-caves		2021	vessel	6	2^a^	2	E	GF, 1.5	Silica beads	BTT	100	8
BDA-diving transect			diving	7	4	0.12–0.14	S	GF, 1.5	Silica beads	BTT	100	8
GSL-2020	Estuary and Gulf of St Lawrence	2020	vessel	59	1	2	V	GF, 1.2	Frozen	BTT	80	3
GSL-2021		2021	vessel	46	1	2	E	GF, 1.5	Silica beads	BTT	80	3
GSL-2022		2022	vessel	86	1	2	E	GF, 1.5	Silica beads	MN	100	3

PES: polyethersulfone, GF: glass fiber, BTT: QIAGEN DNeasy blood and tissue DNA extraction kit, MN: Macherey-Nagel nucleospin tissue extraction kit, V: vacuum pump in the lab; E: Smith-Root eDNA sampler; S: syringe. ^a^Except for station 16 where 1 m sample was not collected.

**Fig. 3:**
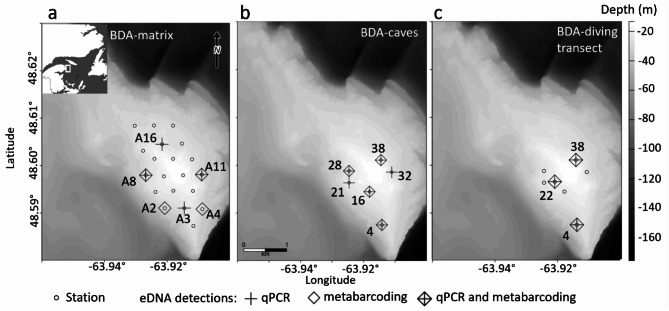
Fine scale experimental designs and eDNA detections for *Anarhichas lupus* at the Banc-des-Américains Marine Protected Area (BDA-MPA) in the BDA-matrix (two samples per station) (**a**), BDA-caves (two samples per station) (**b**) and BDA-diving transect (**c**). Geographic reference system: NAD83 (CSRS) Quebec Lambert, EPSG 6622. Raster files were sourced from the Canadian Hydrographic Service (Canadian Hydrographic Service Non-Navigational (NONNA) Bathymetric Data - Open Government Portal). Only stations with detections have a name in the figure, for future monitoring efforts. All stations coordinates are provided in supplementary material.

### Laboratory analyses

All samples and filters were processed in a suite of dedicated eDNA ultraclean laboratories with external filtered air (HEPA, high-efficiency particulate air). Filtration, extraction, and PCR preparation were done in separate rooms. Laboratory users were trained to work in clean conditions according to standard operating procedures for eDNA (e.g., specific instructions about when to wear and change sterile gloves, coats, mobcaps, chirurgical masks, and overshoe protections). Rooms were also decontaminated once a week with a 0.6% sodium hypochlorite solution (see^33^ for more details).

Frozen water samples were thawed overnight and then filtered with a vacuum pump system (Table 1). A filtration negative control with Milli-Q^®^ water was included on each filtration day. Filters were preserved at -20 °C until DNA extraction.

Filters were cut in halves prior to DNA extraction. DNA was extracted from one half with optimized DNeasy^®^ Blood and Tissue DNA extraction kit (QIAGEN, Germantown, MD, USA) or the NucleoSpin Tissue kit (Macherey-Nagel, Düren, Germany)^34,35^. Remaining filter halves were frozen for long term storage at -80 °C. An extraction negative control (filter immerged in Milli-Q^®^ water) was included with the samples per extraction batch. DNA extracts were preserved at -20 °C prior to qPCR detection or library preparation.

### Single-species detection

We tested each DNA extract with a minimum of three qPCR replicates for the *A. lupus* single-species qPCR assay (Table 1) and with one qPCR replicate for the inhibition qPCR assay^26^. Each qPCR plate contained 89 to 91 DNA extracts, one to five negative controls, and one positive control (i.e., synthetic sequence of the species of interest with an extra 6-nucleotide to identify suspected laboratory contamination). A negative control failed if a PCR amplification was observed, and a positive control failed if the amplification was delayed by > 2 Cq.

### Multi-species detection

Metabarcoding analyses were performed in three distinct batches, with slight variation on the loci targeted and methods used (Table 2).

**Table 2:** Summary information of bioinformatics analyses and sequencing depth obtained from metabarcoding assays across datasets and treatment years. BDA-MPA = Banc-des-Américains marine protected area; GSL = gulf of St. Lawrence.

Treatment year	Dataset (*n* = number of samples)	Assay	DADA2 version	Mean reads per sample ± standard deviation
2020	BDA-MPA-2020 (*n* = 40)	MiFishU^36^	1.22.0	206,882 ± 279,164
2021	GSL-2021 (*n* = 43)	MiFishU^36^, 12S248^37^	1.24.0	MiFishU: 110,565 ± 39,375 12S248: 32,518 ± 17,632
2022	GSL-2020 (*n* = 59)	MiFishU^36^, 12S160^38^	1.28.0	MiFishU: 317,973 ± 198,714 12S160: 384,407 ± 194,133
	GSL-2022 (*n* = 86)			MiFishU: 321,781 ± 166,737 12S160: 311,790 ± 198,177
	BDA-MPA-2021 (*n* = 38)			MiFishU: 239,309 ± 261,352 12S160: 238,572 ± 228,814

The 12 S mitochondrial DNA assays used were the MiFishU (~ 170 bp^36^), 12S160 (~ 115 bp^38^), and 12S248 (~ 160 bp^37^). Libraries were prepared with two rounds of PCRs by either Genome Quebec with combinatorial dual indexing (BDA-MPA-2020) or at MLI with unique dual indexing (BDA MPA-2021, GSL 2020–2022) following the protocol from^37^. Each PCR plate contained two negative controls (Milli-Q water), and one qualitative positive control (a sample previously processed and showing detection results). Libraries were sequenced on an Illumina NovaSeq PE 250 at Genome Quebec.

Reads were demultiplexed by the sequencing facility, and quality control of raw reads was assessed with FastQC^39^ and MultiQC^40^. Adapters were retrieved and removed with cutadapt^41^. The DADA2 R package^42^ was used to filter and merge reads, create exact sequence variants (ESV), remove chimeras and compile ESV tables (Table 2). Taxonomic assignments were performed with the BLAST + tool blastn v2.12.0^43^, using a Top Hit approach at the 95% identity threshold^44^ on NCBI nucleotide database^45^. MetabaR v1.0.0^46^ was used to check and correct potential tag-jumping. Bioinformatic treatment was implemented using in-house R pipeline v0.1.0 (https://github.com/GenomicsMLI-DFO/MLI_metabar_pipeline). We computed mean sequencing depth for each assay and sequencing batches. Negative controls failed if *Anarhichas* reads were detected.

### Statistical analyses and data visualization

All statistical analyses were performed with the R environment^47^. We compared *A. lupus* detections methods using eDNA (i.e., qPCR, metabarcoding) or visual observations (i.e., trawling, diving) with Venn diagrams (*ggVennDiagram* R package). Spearman Rank Correlations were used for pairwise testing of the relationships between qPCR mean copy number per sample, metabarcoding reads count per sample (log transformed), and biomass estimates (*stats* R package). To account for the potential effect of stations (BDA-matrix and BDA-caves datasets only) we randomly selected one observation per station and performed a bootstrap analysis with 999 iterations to compute a mean rho and a 95% confidence interval (CI). Statistical significance of the sampling year (factor), number of reads (for metabarcoding model only), and biomass estimates, and their interaction with year, on the probability of *A. lupus* detection (0 or 1) was assessed with a generalized linear model (logit link and binomial error structure) (*stats* R package). Final models were selected with backward variable selection.

## Results

A total of 301 water samples were collected from 215 stations between 2020 and 2022 (Fig. 1, Supplementary Table S1). All qPCR and metabarcoding negative controls were successful, confirming that no contamination was detected in field, extraction, or amplification negative controls. Positive controls also had expected results for the qPCR and metabarcoding assays (see methods for criteria). Furthermore, no inhibition was detected in any samples.

The mean sequencing depths of metabarcoding assays showed relative consistency in the 2020 and 2022 metabarcoding batches across surveys and assays (between 206,882 and 384,407 reads) but lower numbers of reads were observed in the 2021 GSL metabarcoding batch (110,565 reads for MiFishU, 32,518 reads for 12S248; Table 2).

### Estimating reliability and dispersion of eDNA in fine scale surveys

A total of 27 stations were successfully sampled in the fine scale surveys of the ridge on the Banc-des-Américains Marine Protected Area (BDA-MPA, Table 1): (1) 20 stations separated by 450 m (BDA-matrix) (Fig. 3a), (2) six stations over *A. lupus* occupied caves (BDA-caves) (Figs. 2a and 3b), and (3) seven caves with perpendicular transects (BDA-diving transect) (Figs. 2b and 3c).

Detection rates of eDNA varied greatly between the BDA-matrix and BDA-caves but varied slightly between the qPCR and MiFishU metabarcoding assays (Fig. 3a and b). For the BDA-matrix, *A. lupus* DNA was detected with the qPCR assay in 20% of the stations (*N* = 4) and detections ranged between 7.0 and 51.9 DNA copies per qPCR reaction (Fig. 3a). Similarly, *Anarhichas* DNA was also detected in 20% of stations (*N* = 4) with read counts ranging from 636 to 64,992 (representing 8.9 to 15.1% of reads per reaction) with the MiFishU assay (Supplementary Table S1). Only two stations had eDNA detected with both assays (Fig. 3a). *Anarhichas* DNA was detected in 30% of stations when results from the single- and multi-species assays were combined. For the BDA-caves, eDNA was detected in all stations (*N* = 6) with the qPCR assay and in 67% of stations (*N* = 4) with the MiFishU assay (Fig. 3b). Detections of eDNA ranged between 1.2 and 9.0 DNA copies per qPCR reaction and between 2,281 and 24,904 read counts (representing 0.6 to 4.6% of reads per reaction) for the MiFishU assay (Supplementary Table S1). Correlations between the qPCR copy numbers and the MiFishU read counts were low with the BDA-matrix (mean rho = 0.19, 95% CI [-0.14, 0.73], *N* = 20 stations) and moderate with the BDA-caves (mean rho = 0.38, 95% CI [-0.14, 0.89], *N* = 6 stations).

Detection rates of eDNA per station were greater when combining results from samples collected at 1 and 2 m from the sea floor in BDA-matrix and BDA-caves. For BDA-matrix, 100% eDNA detections (*N* = 4) by qPCR and MiFishU metabarcoding per station relied on a single sample either collected at 1–2 m above the sea floor (Supplementary Table S1). For BDA-caves, half of qPCR (50%, *N* = 3) and all the MiFishU metabarcoding (100%, *N* = 4) eDNA detections per station relied on a single positive detection either in the 1–2 m sample (Fig. 2a, Supplementary Table S1).

**Fig. 4:**
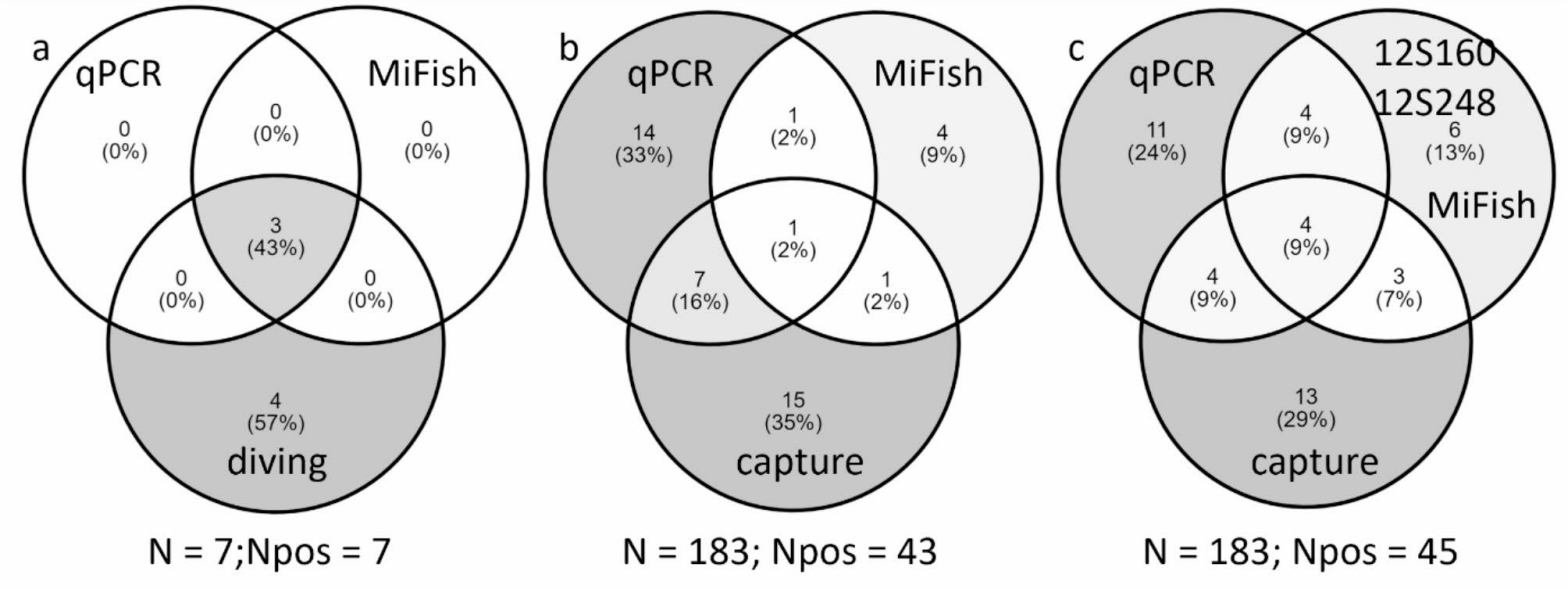
Relations between detections or observations of *Anarhichas lupus* or *Anarhichas* spp. among methods used (i.e., diving observations or trawl captures, qPCR, and metabarcoding assays) in (**a**) BDA-diving transect and (**b**, **c**) GSL surveys. Panels (**a**) and (**b**) include results from the MiFishU metabarcoding assay only while panel (**c**) also included 12S160 and 12S248 metabarcoding assay results. Numbers and proportions (in parenthesis) of positive stations where *A. lupus* (qPCR, trawl captures, and diving) or *Anarhichas* spp. (metabarcoding assays) eDNA was detected or observed given a specific method are presented. N represents the total number of stations sampled and N_pos_ the number of positive stations.

In BDA-diving transect, highly localized eDNA detections were observed at the entrance of the cave lodging one or two wolffish (Supplementary Table S1). The eDNA detection rates either with the qPCR and MiFishU assays were 43% (*N* = 3) for stations at 0 m and 0% for stations at 5 to 15 m along perpendicular transects from caves, with 100% (*N* = 3) consistent detections between both assays at positive stations (Figs. 2b, 3c, 4a). Detections of eDNA ranged from 2.0 to 7.0 DNA copies for the qPCR assay and between 37,141 and 333,408 read counts (representing 16.9 to 65.7% of reads per reaction) for the MiFishU assay (Supplementary Table S1). Correlation between the qPCR copy numbers and the MiFishU read counts was high in BDA-diving transect (rho = 0.99, *P* < 0.001, *N* = 27 samples).

### Combining eDNA detections and visual observations in the broad scale surveys

One water sample was collected at each of the 188 stations of the broad scale survey in the Estuary and Gulf of St. Lawrence (GSL) over the three years (N_2020_ = 59, N_2021_ = 43, N_2022_ = 86), and eDNA was assessed for *A. lupus* with the qPCR assay and for *Anarhichas* with metabarcoding assays in all samples collected (Fig. 1). Trawl captures were obtained in 98% of eDNA stations (*N* = 183; 100% in 2020, 98% in 2021, 96% in 2022).

**Fig. 5:**
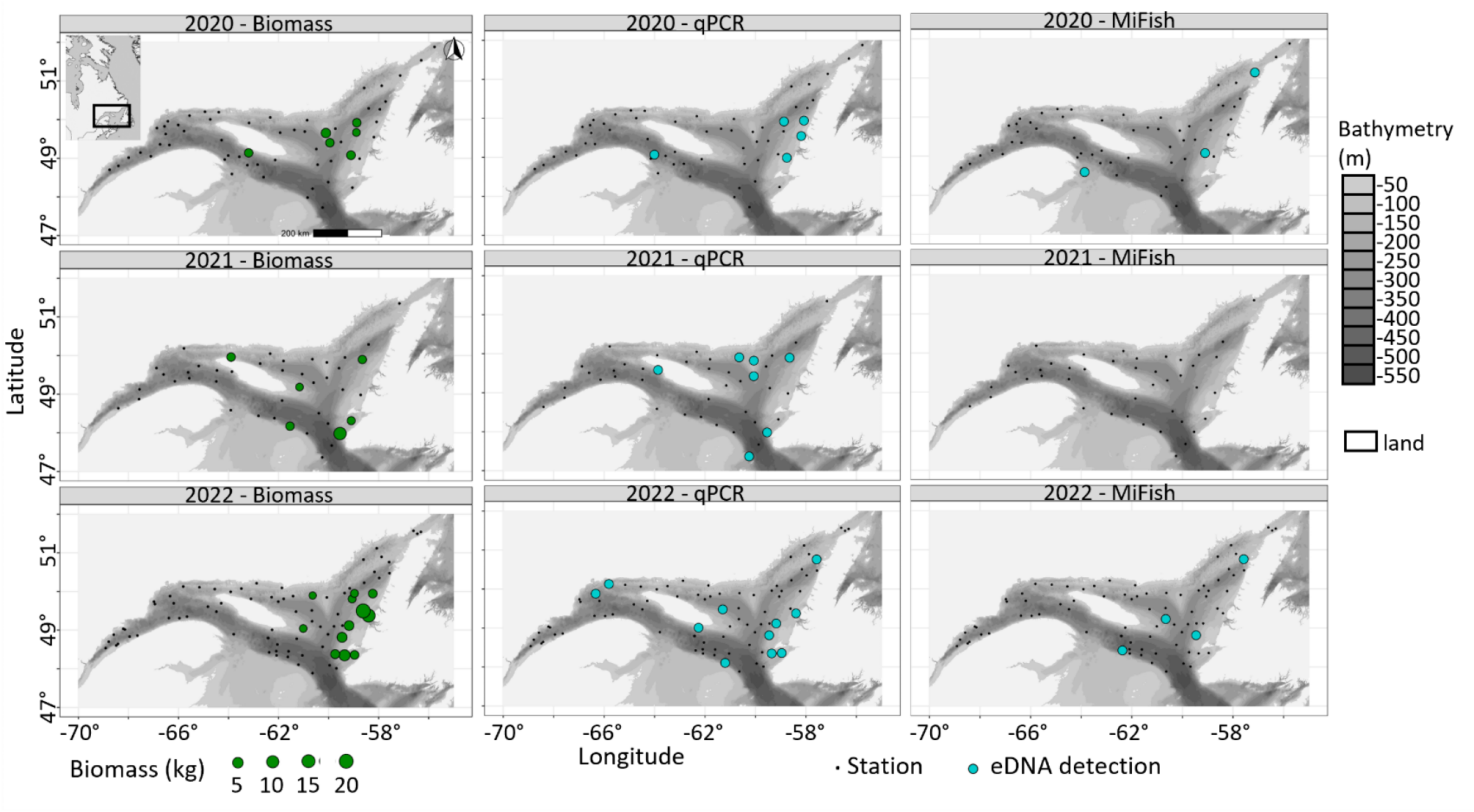
Broad scale experimental design in the Estuary and Gulf of St. Lawrence showing biomass, qPCR, and metabarcoding detections for *Anarhichas lupus* and surveys conducted from 2020 to 2022. Geographic reference system: NAD83 (CSRS) Quebec Lambert, EPSG 6622. Raster files were sourced from the National Oceanic and Atmospheric Administration (Bathymetric Data Viewer). Details of positive stations are provided in Supplementary Table S1.

Detection rates of *A. lupus* with the single-species assay were greater than those for *Anarhichas* DNA with the multi-species assay in the GSL for all three years (Figs. 4b and 5). DNA was detected in 12% (*N* = 23) and 4% (*N* = 7) of stations sampled across the three years with the qPCR and the MiFishU assays, respectively (Supplementary Table S1) and in 15% of stations when the two assays were combined (qPCR and MiFishU). With the qPCR assay, *A. lupus* DNA was detected every year in low proportions of GSL stations, i.e., 8% in 2020 (*N* = 5; 10.0 to 21.0 DNA copies per qPCR reaction), 16% in 2021 (*N* = 7; 2.0 to 5.9 DNA copies per qPCR reaction) and 13% in 2022 (*N* = 11; 1.5 to 20.2 DNA copies per qPCR reaction; Fig. 5, Supplementary Table S1). No difference was observed in the probability of detection among GSL surveys or years (chi^2^ = 1.48, d.f. = 2, *P* = 0.48, *N* = 188). Similarly, *Anarhichas* DNA was detected every year in low to null proportions of stations with the MiFishU assay, i.e., 5% in 2020 (*N* = 3; 7,008 to 42,688 reads or 3.4 to 18.3% of reads per reaction), 0% in 2021, and 5% in 2022 (*N* = 4; 168 to 1,076 reads or 0.04 to 0.2% of reads per reaction; Fig. 5, Supplementary Table S1). Again, no differences were observed in the probability of detection among surveys (chi^2^ = 3.73, d.f. = 2, *P* = 0.15, *N* = 188) or sequencing depths (chi^2^ = < 0.01, d.f. = 1, *P* = 0.93, *N* = 188). Correlations were null or low between qPCR copy numbers and MiFishU read counts per survey (2020: rho = -0.07, *P* = 0.60, *N* = 59; 2022: rho = 0.11, *P* = 0.007, *N* = 86).

We also investigated in a subset of years the ability of two other metabarcoding assays, namely the 12S160 and 12S248 (Table 2, Supplementary Fig. S1), to detect *Anarhichas* DNA, and higher detections rates were observed some years compared to the MiFishU assay. With the 12S160 assay, *Anarhichas* DNA was detected in 0% stations sampled in 2020 and 9% stations sampled in 2022, which is lower and higher, respectively, than the detection rate of the MiFishU assay (Supplementary Table S1 and Fig. S1). Read counts of *Anarhichas* DNA per sample with the 12S160 metabarcoding assay ranged from 2,349 to 121,960 for 2022 samples (representing 1.2 to 32.6% reads per reaction). With the 12S248 assay, *Anarhichas* DNA was detected in 12% of stations (*N* = 5) sampled in 2021, which is higher than with the MiFishU assay (Supplementary Table S1 and Fig. S1). Read counts of *Anarhichas* DNA per sample with the 12S248 metabarcoding assay ranged from 382 to 15,323 (representing 0.8 to 23.8% reads per reaction). Correlations between copy numbers detected by qPCR and read counts for the 12S160 and the 12S248 assays were moderate and greater than that with the MiFishU assay, i.e., rho = 0.49 for 12S248 in 2021 (*P* < 0.001, *N* = 43) and rho = 0.40 for 12S160 in 2022 (*P* < 0.001, *N* = 86).

There were low to moderate overlap between trawl *A. lupus* captures and eDNA detections with the qPCR assay and each of the three metabarcoding assays (Figs. 4b and 5, Supplementary Table S1). Specimens of *A. lupus* were captured in 13% (N = 24) out of the 183 trawls during the three years sampling of the GSL, corresponding to 10% (N = 6) of stations in 2020, 14% (N = 6) of stations in 2021, and 15% (N = 12) of stations in 2022. From the 183 stations with trawl captures and eDNA detections (qPCR, MiFishU metabarcoding), 23% stations (N = 43) had *Anarhichas* captured or detected with eDNA (Supplementary Table S1). Presence of *A. lupus* or inference of *Anarhichas*’ presence in the 43 stations occurred exclusively with captures in 35% of stations (*N* = 15), with the qPCR detections in 33% of stations (*N* = 14), and with the MiFishU detections in 9% of positive stations (*N* = 4) (Fig. 4b). The trawl captures and the qPCR detections represented 91% (*N* = 39) of *A. lupus* observations or detections over all three years surveyed with the MiFishU detections (Fig. 4b). Similarly, most observations or eDNA detections were due to a combination of trawl captures and qPCR detections each year or across years with the two other metabarcoding assays, i.e., 12S160, 12S248 (Supplementary Fig. S1). Combining the detection results of the three metabarcoding assays also increased the proportion of exclusive detections with this approach from 9% (*N* = 4, MiFishU) to 13% (*N* = 6) of positive stations (Fig. 4b and c). Still, most detection or observation were due to the combination of trawl captures and qPCR detections considering the three metabarcoding assays (87%, Fig. 4c). Note that the *Anarhichas* detection rate was 25% of stations (*N* = 47) after combining trawl captures, eDNA detections by qPCR and three metabarcoding assays (MiFishU all years, 12S160 in 2020 and 2022, 12S248 in 2021) (*N* = 188, Supplementary Table S1).

Correlations between biomass estimates from the trawl and eDNA detections were highly variable across assays and years. The biomass of captured specimens ranged between 0.01 and 20.57 kg overall years, and between 0.11 and 2.08 kg in 2020 (N_specimens_= 24), 0.06 to 11.92 kg in 2021 (N_specimens_ = 32), and 0.01 to 20.57 kg in 2022 (N_specimens_= 116) (Fig. 5, Supplementary Table S1). Probability of qPCR detections increased with the biomass estimates (chi^2^ = 6.78, d.f. = 1, *P* = 0.009, *N* = 183), with a positive moderate correlation between qPCR copy numbers and biomass estimates (rho = 0.27, *P* < 0.001, *N* = 183; Supplementary Fig. S2). In contrast, the probability of MiFishU detections did not increase with the biomass estimates (chi^2^ = 0.065, d.f. = 1, *P* = 0.80, *N* = 183), and read counts and biomass estimates were not correlated (rho = 0.10, *P* = 0.17, *N* = 183). Correlations between biomass estimates and read counts were moderate for the 12S160 assay (2022: rho = 0.36, *P* < 0.001, *N* = 82) and 12S248 assay (2021: rho = 0.51, *P* < 0.001, *N* = 42).

## Discussion

Our study showed that (1) detections of wolffish eDNA were reliable and highly localized with the fine scale experimental designs at the BDA-MPA, (2) the species distribution can be improved by combining trawl captures and eDNA detections with the broad scale experimental design in the GSL, and (3) the qPCR assay is more sensitive than the three metabarcoding assays tested at the fine and broad scales. Overall, our results suggest a clear improvement in the description of a rare marine species distribution using eDNA and that this method should be integrated to marine surveys. We will also present possible improvements to eDNA detections based on our results.

### Reliable and highly localized eDNA detections of rare species

The BDA-matrix had 30% positive stations while the BDA-caves had 100% positive stations using eDNA from the combined results of the qPCR and the metabarcoding assays in the BDA-MPA (Fig. 3a and b). While the BDA-matrix confirms the rarity of the species in the area, the BDA-caves shows the high reliability of species inference using eDNA detections and oceanographic sampling methods. Our unique experimental design, coupling Niskin bottle sampling over diving observations of a rare species, allowed to test in the natural environment the reliability of eDNA detection methods in samples collected during oceanographic surveys. Previous studies had shown with water sampling and observations from divers that eDNA detections are reliable and complementary to visual surveys (e.g., red handfish, *Thymichthys politus*^48^, Pacific rockfish species^49^), but few of these studies had evaluated the reliability of eDNA detections with water collected with an oceanographic method^50,51^.

The BDA-diving transect showed that eDNA detections of Atlantic wolffish were limited to the cave entrance, indicating the limited dispersal of eDNA for rare species. Our results match those of the rare red handfish (*Thymichthys politus*) with ca. 100 adults remaining in the wild that are inhabiting shallow vegetated coastal habitat on and adjacent to fringing rocky reefs^48^. In their study, 10 L water samples were collected at various distances along transects from a small agglomeration^48^. Detections of red handfish eDNA only occurred at 0 m (1/9 or 1/3 positive qPCR replicate) from specimens. Other studies have shown greater distances of DNA dispersion in other marine systems but these studies were using species with higher abundance than those of rare species. For instance, water collected along different distances from salmonids farms showed that eDNA detections may occur as far as ca. 1.5 km from the source in two marine coastal environments^13,14^. In an experimental design with less fish, eDNA was detected up to 100 m from a caged fish experiment with 49 striped jack (*Pseudocaranx dentex*) in the Maizuru Bay (Sea of Japan^15^). At the multi-species level, a study showed that taxa similarities between communities, determined from eDNA, decreased with physical distance, implying that the effectively sampled area for eDNA is less than 100 m in a nearshore environment^16^. The very small detection distance reported in our BDA-diving transect survey is likely due to the fact that either one or two wolffish inhabit a cave in boulder bedrocks. *Anarhichas lupus* displays a largely sedentary and solitary life^17^ limiting the production of eDNA to small DNA concentrations. Aquaculture farms or species moving in shoal most likely produce more eDNA and DNA transport over longer distance may be an issue to estimate precisely the position of the animals. The highly hydrodynamic ecosystem of the BDA-MPA, particularly on the ridge^28^, may also drive the rapid dispersion of DNA^52^. These biological and physical conditions lead to a DNA concentration high enough for detection only close to *A. lupus* cave (≤ 2 m) limiting the potential for false positives (e.g. eDNA transport). Our work with this rare and solitary species supports previous hypotheses that eDNA detections are highly localized in a coastal marine environment^15^.

### The combination of eDNA detections and visual observations improve the characterization of a rare species occurrence

Captures from trawl surveys and eDNA detections are hardly comparable since bottom-trawls are integrated over space and time whereas eDNA is instantaneous^53,54^. The spatial area covered by one tow in the GSL surveys is 0.0684 km^2 53^ but the worldwide average is ca. 9 km^2^ (2001–2019 datasets^55^). In contrast, eDNA detections often provide a highly localized inference of species presence using a Niskin bottle^15^. The distinctiveness of these survey methods also explains why their combination is an effective approach to improve the distribution of rare marine species. Our results showed that the combination of eDNA detections (MiFishU metabarcoding and qPCR) and trawl captures from the broad scale survey increased from 13% (trawl captures only) to 23% of stations (trawl captures, qPCR, MiFishU metabarcoding detections) the occurrence frequency of Atlantic wolffish. Multiple previous studies showed that the combination of traditional observations and eDNA detection methods is improving common marine species occurrence data^7,8,53,56^. The 10% increase of frequency of occurrences may be meaningful to improve the monitoring of a species with a conservation status in Canada and other countries. Detections of eDNA may also help to monitor the occurrence of other species inhabiting boulder bedrocks or complex sea bottoms such as relatively high surface curvature and high substrate coarseness, which are associated to untrawlable habitats^1^. Examples of such fish species in the North Atlantic include the ocean pout (*Zoarces americanus*), the radiated shanny (*Ulvaria subbifurcata*), the slender eelblenny (*Lumpenus fabricii*), the banded gunnel (*Pholis fasciatus*) and rock gunnel (*P. gunnellus*).

The occurrence frequency using eDNA and/or trawl for stations on the ridge of BDA-MPA was 30% (qPCR and MiFishU metabarcoding), and in the GSL was 23% (qPCR, MiFishU metabarcoding, trawl). Those occurrence frequencies are high for an endangered species, at least higher than that of other species at risk yet tested in the marine environment. The critically endangered European eel (*Anguilla anguilla)* was detected with eDNA metabarcoding in 9% of MPA stations sampled over multiple years^57^. Similarly, the critically endangered elasmobranch *Pristis pectinata* was detected with eDNA qPCR in 7% stations from three areas in the Gulf of Mexico^58^. It is clear from our results that the higher rates of positive detections for *A. lupus* in the BDA-MPA and GSL is partially explained by the combination of at least two methods (e.g., eDNA single-, multi-species, trawl). The sampling within the BDA-MPA already known for the presence of Atlantic wolffish may also partially explain the high frequency of occurrence in this area. Still, our results suggest that a combination of monitoring methods including eDNA detections improves the characterization of rare species frequency of occurrence, which could suggest a reassessment of their conservation status. The combination of trawl captures and eDNA detections in the broad scale survey would also allow for more precise monitoring of the species on an annual basis, which is a desirable outcome for species with a conservation status.

### Comparison of assays’ performance and future improvements of eDNA detection methods

Our eDNA results with the qPCR and the metabarcoding assays agree with the solitary life mode of Atlantic wolffish. The mean copy numbers estimated with the qPCR assay or the proportions of reads from the metabarcoding assays at positive stations were low and similar between the fine and broad scale surveys. This was expected based on biomass estimates and the frequency of occurrence observed in bottom-trawl surveys due to the sporadic capture of these animals^30–32^.

In contrast, the qPCR assay was more sensitive for the detection of *A. lupus* eDNA than the metabarcoding assays in the fine and broad scale surveys. In BDA-caves, an important proportion of eDNA detections (33%) were only achieved with the qPCR assay (Fig. 3b). A similar pattern was observed with the GSL surveys where all metabarcoding assays produced fewer detections than the qPCR assay (Fig. 5, Supplementary Fig. S1). Our results confirm those from previous studies indicating that the qPCR is more sensitive than multi-species metabarcoding^59^. This difference in sensitivity between eDNA methods can be explained by the use of a higher number of replicates in qPCR than in metabarcoding^16,60,61^ and primer bias in multi-species metabarcoding^62^. Recent studies also suggested that, in addition to using replicates in metabarcoding studies, employing a single-species metabarcoding assay can enhance specificity and sensitivity. This method can even exceed qPCR results in detecting crab and fish species^63,64^ but at a higher cost than the qPCR analyses used in our study.

Combining detections from several metabarcoding assays is an alternative way to increase the sensitivity of the method^65,66^. In our study, the combination of detections from the three metabarcoding assays increased from 9 to 13% the frequency of eDNA detections across the 188 stations which is similar to the 12% detection rate of the qPCR assay (see results). The performance of some metabarcoding assays is likely underestimated in our study due to the low sequencing depths of some assays (e.g., 12S248). An increase of the sequencing depth in metabarcoding assays generally benefit low-abundance species detection^61,62^. Also, metabarcoding assays used in our study target the same mitochondrial gene (12 S) for fish detection, therefore the gain in detection rate from multiple assays could be lower in comparison to the use of assays targeting different mitochondrial genes, e.g., 16 S, cytochrome c oxidase I (COI), and 12 S^66^.

Increasing the number of sample replicates collected may also improve the detection rate with any assays. Correlations between qPCR copy numbers and MiFishU read counts lacked when one sample was tested in the broad scale survey whereas high correlations were observed when two samples were tested in the fine scale surveys. With the fine scale surveys, our results also suggest that eDNA detections from one sample may not be sufficient to detect rare species eDNA. Our results are coherent with the general observation that increasing the sampling effort help to improve detection frequency^66–68^. This may be even more important for rare species.

We also tested for the correlation between biomass and eDNA detections to investigate further results obtained in this study. Poor to high correlations were observed between biomass and qPCR or metabarcoding detections. The 12S248 assay had the highest correlation coefficient with biomass compared with the MiFishU and 12S160 assays. While the direct comparison between eDNA and trawl survey may be biased because of the contrasting area sampled with the two methods^15,55^, other studies have also identified positive relationships between metabarcoding eDNA reads and abundance or biomass for relatively abundant marine species^69,70^. The stochasticity of bottom-trawl capture (i.e., varying catchability) or eDNA detections for rare species may explain the weak correlation between these two methods^12,71^. Some of this variability may also be due to the primer bias towards some species DNA. More research should be pursued to discriminate the effect of these factors in the natural environment on eDNA detections.

### Implications for marine protected area monitoring

Overall, our eDNA detection data is increasing *A. lupus* frequency of occurrence in the GSL which improves data available for the reassessment of its conservation status in Canada. We also want to highlight that although the species may be more frequent than expected at both scales, our eDNA results agree with the solitary life mode of this species and its heterogenous distribution. In Marine Protected Areas, eDNA studies gain in popularity notably to inform marine spatial planning decisions^57,68,72,73^. In terms of conservation and resource management, our study shows that there is a clear benefit to complement traditional monitoring with environmental DNA for Species-at-Risk conservation. The prioritization in the selection of new MPAs needs to consider endangered species distributions^74^. eDNA detections for *A. lupus* in the GSL could also play a major role in the choice of locations of new MPAs, created to reach the international target of 30% of the global ocean protected in 2030.

## Electronic supplementary material

Below is the link to the electronic supplementary material.

Supplementary Material 1

Supplementary Material 2

## Data Availability

Data is provided within the manuscript or supplementary information files.
