# Supplementary material for: Improving an endangered marine species distribution using reliable and localized environmental DNA detections combined with trawl captures
Source: Sci Rep. 2025 Apr 8;15:11926. doi: 10.1038/s41598-025-95358-3 (PMC11976931; doi:10.1038/s41598-025-95358-3)
Supplement: Supplementary file 2 — Supplementary Material 2 [file 41598_2025_95358_MOESM2_ESM.docx]

Improving the description of an endangered species distribution using environmental DNA detections combined with trawl captures in the marine environment

Author information:

Marion Chevrinais^1,*^, Audrey Bourret^1^, Geneviève Côté^1^, Geneviève Faille^1^, Nellie Gagné^2^, Geneviève J. Parent^1^

^1^Fisheries and Oceans Canada, Maurice Lamontagne Institute, Mont-Joli (QC), Canada

^2^Fisheries and Oceans Canada, Gulf Fisheries Centre, Moncton (NB), Canada

*Corresponding authors marion.chevrinais@dfo-mpo.gc.ca, genevieve.parent@dfo-mpo.gc.ca

Supplementary material

Table S1. Sampling sites, GPS coordinates in decimal degrees (WGS84: EPSG 4326) and eDNA detections of *Anarhichas lupus.*


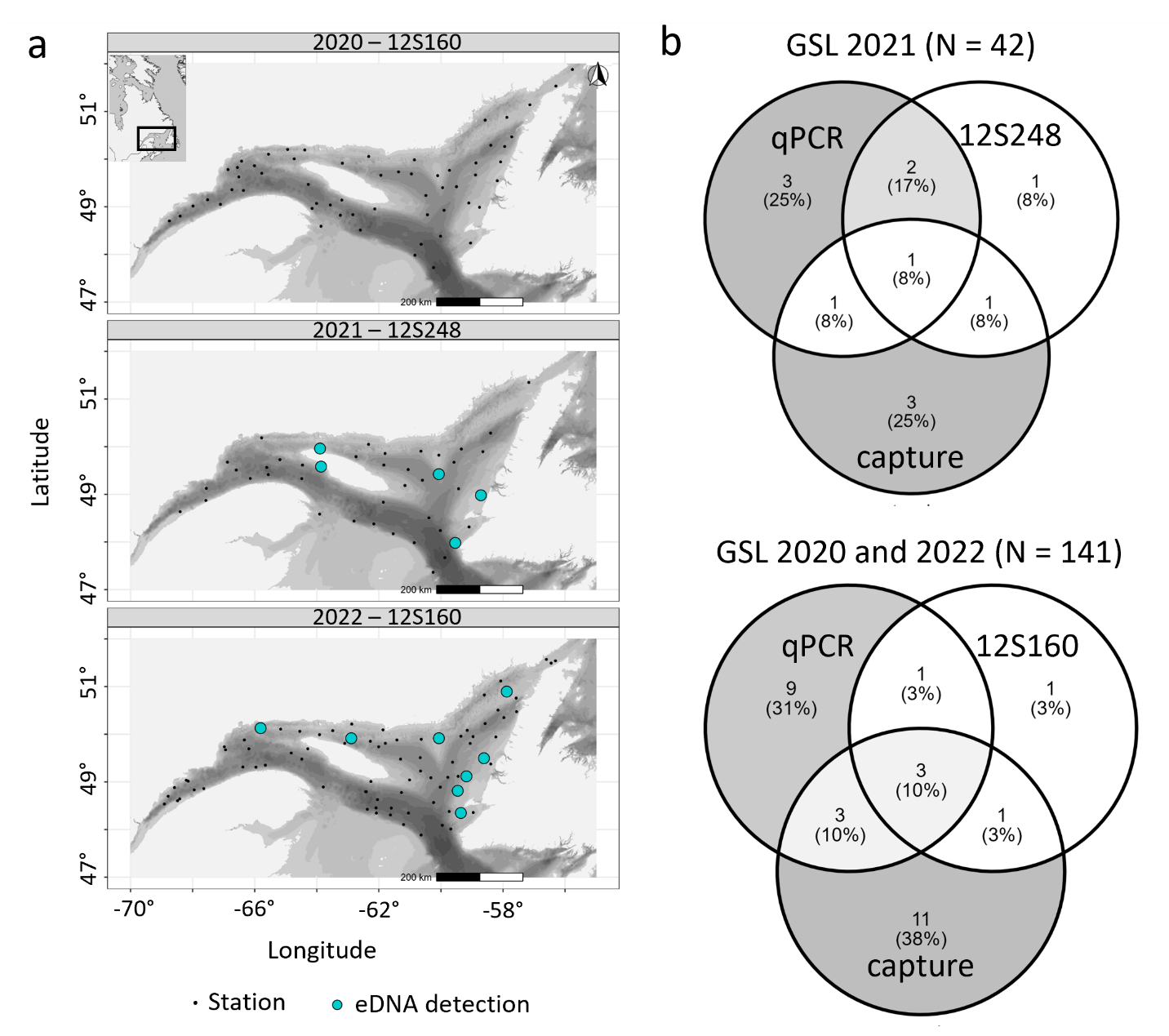


Fig. S1. a. Large scale study area showing 12S160 and 12S248 metabarcoding detections for *Anarhichas* in the Estuary and Gulf of St. Lawrence between 2020-2022. Geographic reference system: NAD83 (CSRS) Quebec Lambert, EPSG 6622, raster files from the National Oceanic and Atmospheric Administration. b. Venn diagrams showing shared *A. lupus* detection in qPCR, metabarcoding and captures. Numbers represent stations where *A. lupus* (or *Anarhichas* for metabarcoding) DNA was detected. Details of positive stations in Supplementary Table S1.


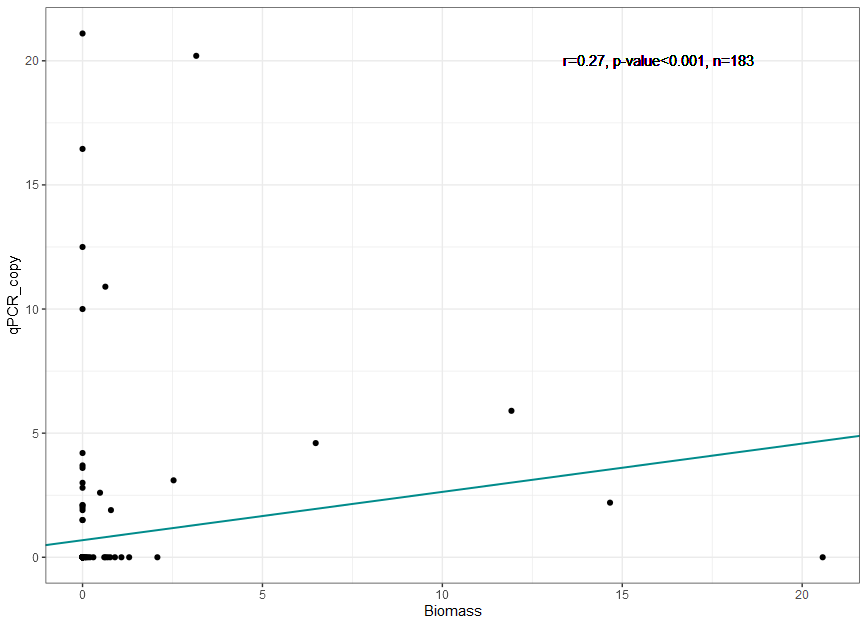


Biomass estimate (kg)

qPCR copy number (copy/reaction)

Fig. S2. Correlation plot of the qPCR copy number and the biomass estimates for the GSL dataset (2020, 2021 and 2022). r is for the rho value, n the number of samples. The line represents the trend of the values based on Spearman rank correlation test.
